# Sudden infant death syndrome — a community intervention project

**DOI:** 10.1590/1984-0462/2024/42/2022205

**Published:** 2024-05-27

**Authors:** Ana Fraga, Aida Correia de Azevedo, Joana Veloso, Marta Ferreira, Filipa Carvalho, Filipa Vale, Ana Correia de Azevedo, Ana Luísa Corte-Real

**Affiliations:** a Hospital de Santo André, Centro Hospitalar de Leiria, Serviço de Pediatria, Leiria, Portugal.; b Hospital São João de Deus, Centro Hospitalar do Médio Ave, Serviço de Pediatria, Vila Nova de Famalicão, Portugal.; c CeS Ave/Famalicão, USF S. Miguel-O-Anjo, Vila Nova de Famalicão, Portugal.; d ACeS Ave/Famalicão, USF Ribeirão, Vila Nova de Famalicão, Portugal.; e ACeS Ave/Famalicão, USF Famalicão I, Vila Nova de Famalicão, Portugal.; f ACeS Ave/Famalicão, USF Joane, Vila Nova de Famalicão, Portugal.

**Keywords:** Sudden infant death, Infant, newborn, Prenatal education, Early educational intervention, Childcare, Morte súbita do lactente, Recém-nascido, Educação pré-natal, Intervenção educacional precoce, Cuidado da criança

## Abstract

**Objective:**

To capacitate pregnant women to comply with measures designed to prevent sudden infant death syndrome.

**Methods:**

A quasi-experimental study was conducted before and after the intervention that included pregnant women attending the Course of Preparation for Childbirth and Parenthood of Health Centers Cluster. Six training sessions were given in the context of preventing this syndrome. Three questionnaires were applied, one to evaluate the knowledge of pregnant women before classes, other was submitted after the sessions, and another, one month after the birth of the babies, to identify what skills were acquired and which were practiced.

**Results:**

Among 77 studied pregnant women, 70 answered pre-session questionnaire and the proportion of correct answers varied from from 60.0% to 84.3%. After the intervention, 64 women answered the questionnaire and the proportion of correct answers varied between 79.7% and 100% . Prior to the intervention, the most wrong answers were related to the role of smoking as a risk factor for sudden infant death syndrome and to the use of pacifiers as a protective measure. After the sessions, all women answered correctly to the questions concerning where the baby should sleep and the safest way to lay the baby in the cradle.

**Conclusions:**

Health education with the aim of establishing measures may have a significant impact in terms of care delivery and mortality rate caused by sudden infant death syndrome.

## RESUMO

**Objetivo:**

Capacitar as grávidas para o cumprimento de medidas de prevenção da síndrome de morte súbita do lactente.

**Métodos:**

Realizou-se um estudo quase-experimental pré- e pós-intervenção que integrou as grávidas que frequentavam o Curso de Preparação para o Parto e Parentalidade do Agrupamento de Centros de Saúde. Foram ministradas seis sessões formativas no âmbito da prevenção desta síndrome. Três questionários foram aplicados, um para avaliar os conhecimentos das gestantes antes das aulas, outro foi submetido após as sessões, e outro, um mês após o nascimento dos bebês para identificar que conhecimentos foram adquiridos e quais foram praticados.

**Resultados:**

Da amostra de 77 grávidas, relativamente ao questionário pré-sessão (n=70), obteve-se uma proporção de respostas corretas entre 60,0–84,3%. Posteriormente à intervenção (n=64), verificou-se um incremento dos conhecimentos com 79,7–100% de acertos. Previamente à intervenção, as respostas mais erradas às questões eram inerentes ao papel do tabagismo como fator de risco para síndrome de morte súbita do lactente e ao uso da chupeta como medida protetora. Após as sessões, todas as mulheres responderam corretamente às questões relativas ao local onde o bebê deve dormir e à forma mais segura de colocar o bebê no berço.

**Conclusões:**

Este projeto demonstrou que a educação para a saúde com o intuito de instituir medidas pode ter um impacto significativo em termos de prestação de cuidados e taxa de mortalidade por síndrome de morte súbita do lactente.

## INTRODUCTION

Sudden infant death syndrome (SIDS) is the leading cause of post-neonatal mortality in the first year of life in developed countries.^1^ Although the risk is less than 1 per 1,000 births, it is 20 times higher than the risk of death in the subsequent 17 years from another cause. Its peak occurs between two and four months, and most cases occur in the first six months of life at home.^2,3^

The identification of risk factors and public health campaigns adopted in numerous countries have led to a large-scale reduction in its incidence. In Portugal, the Directorate-General of Health and the Portuguese Society of Pediatrics have created recommendations to prevent SIDS. In addition, in the Bulletin of Child and Youth Health are inscribed some guidelines that recommend measures to help prevent this syndrome.^4^

SIDS etiology is unknown, but it is believed to be multifactorial, due to an interaction between an individual's predisposition, triggering factors, and a favorable environment for its occurrence.^5^

Regarding protective factors, they are as follows: breastfeeding; updated vaccination schedule; sleeping in a cot, in a room shared with parents with adequate temperature (18–21°C) and correct aeration; and use of pacifiers.^6,7^

Regarding risk factors, they include those inherent to the infant (prematurity and low birth weight; history of apnea; twinness; brother of an infant who was victim of SIDS), mother (age less than 20 years; complications during pregnancy and/or childbirth; smoking habits; substance abuse during pregnancy); and surrounding environment (sleeping in lateral or ventral decubitus; crib not suitable; objects inside the crib; inadequate room temperature and aeration conditions).^4,5^

As there are proven preventive and risk factors, it is urgent to understand why precautionary measures are not being applied. Thus, with the implementation of this project, the authors aimed to capacitate parents to prevent this syndrome, providing basic tools of health literacy regarding SIDS.

## METHOD

A quasi-experimental before and after intervention study was conducted in which all pregnant women who attended the Health Centers Cluster (*Agrupamento de Centros de Saúde* – ACeS) Ave-Famalicão were invited to participate of the Course of Preparation for Childbirth and Parenthood (CPCP).

The study population consisted of all pregnant women who attended this course between September and November of 2021. The intervention project was designed by all the authors in collaboration with the Community Care Unit (CCU) of the ACeS Ave-Famalicão.

The investigation protocol was submitted and approved by the Technical and Health Committee of the ACeS Ave-Famalicão.

The intervention design included a class about SIDS prevention in the online CPCP. This topic was not previously addressed, so the level of knowledge was standardized for all participants.

In the session conducted by the authors, informed consent was given to each participant after a thorough explanation of this intervention project's aim. After acceptance, they answered anonymous pre- and post-intervention questionnaires using Google Forms^®^.

The questionnaire was composed of six questions with multiple-choice answers. During the first month of their infant's lives, the mothers received a new questionnaire in which they selected the measures they had applied during that period, to assess the degree of compliance with SIDS protective measures. The questionnaires are shown in Table 1.

**Table 1:** Applied questionnaires.

**Compliance with measures to reduce the risk of Sudden Infant Death Syndrome – Pre-session**
Infant Process No: ____________
**Demographic Characterization**
Caregiver Data: - Degree of kinship: ___________ - Age:___________ - Schooling:_________________ Infant Data: - Gender: Female ___ Male ___ - Age: ___ months ___ days
**Questionnaire - Select the correct answer**
**Question 1 - Where should the baby sleep?** In the first days the baby must sleep in the same bed as the parents. The baby must sleep in the crib. It is safe for parents to fall asleep with the baby on their lap. A+B.
**Question 2 - How should the baby sleep?** The baby sleeps on its stomach. The baby sleeps on the side due to reflux. The baby sleeps on its stomach.
**Question 3 - For the baby's cradle, what is the most correct option?** The baby's feet touch the bottom of the bed. We must use duvets, pillows, and cap so the baby is not cold. I must put the toys/toys so that the baby does not feel alone.
**Question 4 - Regarding the room temperature, what is the most correct option?** Regardless of temperature we should wear body, pants, and baby-grow. I must cover the baby up to the neck. The ideal temperature of the room where the baby sleeps should be between 18-21°C. In the first days I should use "baby swaddle" for the baby to sleep.
**Question 5 - Regarding security, what is the most correct option?** When the baby sleeps with a pacifier, it must have a chain. I can leave my child sleeping in the holding chair (babycoque) at home. During the first 6 months babies should not share the same room as their parents. The use of pacifier protects against sudden death syndrome.
**Question 6 - Regarding parents’ risk factors, what is the most correct option?** Smoking during pregnancy is a risk factor for sudden death. If the father smokes at home but not in the room it is safe for the baby. As long as the mother cannot smoke near the baby. A+B.
**Compliance with measures to reduce the risk of Sudden Infant Death Syndrome – Post-session nº. 1**
Infant Process No: ____________ **Demographic Characterization** Caregiver Data: - Degree of kinship: ___________ - Age:___________ - Schooling:_________________ Infant Data: - Gender: Female ___ Male ___ - Age: ___ months ___ days
**Questionnaire - Select the correct answer**
**Question 1 - Where should the baby sleep?** In the first days the baby must sleep in the same bed as the parents. The baby must sleep in the crib. It is safe for parents to fall asleep with the baby on their lap. A+B.
**Question 2 - How should the baby sleep?** The baby sleeps on its stomach. The baby sleeps on the side due to reflux. The baby sleeps on its stomach.
**Question 3 - For the baby's cradle, what is the most correct option?** The baby's feet touch the bottom of the bed. We must use duvets, pillows, and cap so the baby is not cold. I must put the toys/toys so that the baby does not feel alone.
**Question 4 - Regarding the room temperature, what is the most correct option?** Regardless of temperature we should wear body, pants, and baby-grow. I must cover the baby up to the neck. The ideal temperature of the room where the baby sleeps should be between 18-21°C. In the first days I should use "baby swaddle" for the baby to sleep.
**Question 5 - Regarding security, what is the most correct option?** When the baby sleeps with a pacifier, it must have a chain. I can leave my child sleeping in the holding chair (babycoque) at home. During the first 6 months babies should not share the same room as their parents. The use of pacifier protects against sudden death syndrome.
**Question 6 - Regarding parents’ risk factors, what is the most correct option?** Smoking during pregnancy is a risk factor for sudden death. If the father smokes at home but not in the room it is safe for the baby. As long as the mother cannot smoke near the baby. A+B.
**Compliance with measures to reduce the risk of Sudden Infant Death Syndrome – Post-session nº. 2 – 1** ^st^ **month of life**
Infant Process No: ____________
**Demographic Characterization** Caregiver Data: - Degree of kinship: ___________ - Age:___________ - Schooling:_________________ Infant Data: - Gender: Female ___ Male ___ - Age: ___ months ___ days
**Questionnaire Mark with a cross (X) the measures you comply with:**
____ In the first few days the baby sleeps in the same bed as the parents. ____ The baby sleeps in the crib. ____ Sometimes I fall asleep with the baby in my arms. ____ The baby sleeps on its stomach. ____ The baby sleeps on the side due to reflux. ____ Baby sleeps on his stomach ____ Baby's feet touch the bottom of the bed. ____ Use duvets, pillows, and cap so the baby doesn't get cold. ____ I have toys/toys in the crib, so the baby doesn't feel alone. ____ Regardless of the temperature of the room I have worn a body, pants and baby-grow. ____ I cover the baby up to my neck. ____ I try that the ideal temperature of the room where the baby sleeps should be between 18-21°C. ____ In the early days I used "baby swaddle" for the baby to sleep. ____ When the baby sleeps with a pacifier it has a chain. ____ Sometimes I leave my son sleeping in the holding chair (babycoque) at home. ____ My baby sleeps in his room. ____ The use of a pacifier protects against sudden death syndrome. ____ I think smoking during pregnancy is a risk factor for sudden death. ____ The father smokes at home but not in the bedroom, so it's safe for the baby. ____ I can smoke as long as I'm not breastfeeding.

The timeframe for this intervention project was from September 2021 to January 2022, when six sessions were held as part of the CPCP, and questionnaires were sent out for answering. Inclusion criteria were defined as all pregnant women who attended the CPCP and agreed to participate in this project. Exclusion criteria were pregnant women who did not attend the CPCP and those who refused to participate in this project.

The questionnaires were designed by the authors based on the current literature and included six measures of SIDS prevention: where and how the baby should sleep, adequate crib characteristics, ideal room temperature, safety measures, and identification of parental risk factors.

For the questionnaire's validation, a pilot sample was used consisting of ten pregnant women from ACeS Ave-Famalicão who did not participate in CPCP and, therefore, were not part of the study population. The pilot study revealed that the questionnaire was easy to understand and approached important features of newborn care. No changes were made to the questionnaire based on the feedback from these patients.

The data was analyzed using Microsoft Office Excel^®^ and Statistical Package for Social Sciences (SPSS^®^) software, and a Pearson SPSS^®^ χ^2^ test was used to compare the values obtained in the different questionnaires. In the interferential analyses, it was considered significant when p≤0.050.

## RESULTS

The study population included all pregnant women who attended the CPCP of ACeS Ave-Famalicão from September 2021 to January 2022 (n=77). Our sample included all pregnant women who responded to the three questionnaires and participated in the sessions (n=70), which was higher than the calculated sample size (n=65) required to obtain a significant sample from the population with a 95% confidence interval (CI).

Collected data included socio-demographic parameters: the mean age of the future mothers was 32 years, standard deviation (±) 4.4 years and 38.6% had completed high school (12^th^ grade of schooling). The gestational age was between the 28^th^ and the 38^th^ week and three days, with the 32^nd^ week of pregnancy being the most frequent gestational age. Table 2 shows the demographic data.

**Table 2:** Socio-demographic characterization.

Variable		Results (n=70)	
		(%)	(n)
Age group			
	20–25	0.14	1
	25–30	32.90	23
	30–35	35.70	25
	35–40	27.10	19
	40–45	0.29	2
	Mean	31.8	
	Median	30.5	
Years of schooling			
	6^th^ year of schooling	0.14	1
	9^th^ year of schooling	0.86	6
	12^th^ year of schooling	38.60	27
	Graduation degree	28.60	20
	Master degree	22.90	16
Gestational age (weeks)			
	28–32	28.60	20
	32–36	55.70	39
	36–39	11.40	8
	Mean	32.6	
	Median	32.0	

A total of 70 answers were obtained for the pre-session questionnaire and 64 for the post-session questionnaire. Table 3 displays the proportion of correct answers to each question in the questionnaires and the improvement achieved between pre-session and post-session results.

**Table 3:** Results of the questionnaire before and after the intervention, questions and correct answers.

Question	Correct answers		Improvement (%)
	Pre-session (%) (n=70)	Post-session (%) (n=64)	
Where should the baby sleep? Must sleep in crib.	84.3 (n=59)	100 (n=64)	15.7 p=0.001
How should the baby sleep? The baby must sleep on his back.	71.4 (n=49)	96.9 (n=62)	25.5 p<0.001
Baby's cradle characteristics Baby's feet should touch the bottom of the bed.	72.9 (n=51)	100 (n=64)	27.1 p<0.001
Room's temperature The ideal temperature of the room where the baby sleeps should be between 18–21°C.	68.6 (n=48)	96.9 (n=62)	28.2 p<0.001
Safety Pacifier use protects against sudden infant death syndrome.	60.0 (n=42)	92.2 (n=59)	32.2 p<0.001
Parents’ risk factors Smoking during pregnancy is a risk factor for sudden death	65.7 (n=47)	79.7 (n=51)	14 p=0.071
All six questions	27.7 (n=18)	71.5 (n=46)	43.8 p<0.001

Before the clinical session, only 60.0–84.3% of correct answers were obtained. However, significant progress was achieved afterwards (14.0–32.2%), with the proportion of correct answers after the session ranging 79.7–100%. In five out of the total six questions the progress was statistically significant (p<0.001).

Before intervention, only 18 pregnant women answered correctly to all the questions in the questionnaire. Post-intervention, 46 women answered correctly to all the questions, which reinforces that the progress between pre-session and post-session answers was statistically significant (p<0.001).

The post-partum questionnaire was sent by e-mail to the mothers during the first month, and a total of 37 answers were obtained. At this stage, mothers’ mean age was 33.0±4.2 years, and the majority completed high school or had a graduation degree. The most common age of newborns on the day the questionnaire was sent was 30 days of life, being 10% of the babies between the tenth and thirteenth day of life, 14.3% between the fourteenth and twenty-ninth day of life, and 28.6% between the thirtieth and forty-fourth day of life.

Overall, the maximum number of incorrect measures selected were three. Only 8.1% (n=3) of mothers chose all the correct measures without any error; and 40.5% (n=15) of participants did not select incorrect measures, despite not fulfilling all the right boxes. The most common incorrect measure selected was "regardless of the room's temperature, the baby wears a body, plus trousers and baby-grow to sleep" (Table 4).

**Table 4:** Analysis of the before and after intervention's questionnaire.

Measures		Answers	
		(%)	(n)
Correct			
	The baby sleeps in the cradle.	89.2	33
	The baby sleeps with the belly up.	91.9	34
	Baby's feet touch the end of the cradle.	59.5	22
	Room's temperature should be between 18–21°C.	86.5	32
	Pacifier's use protects against SIDSs	48.6	18
	Smoking during pregnancy is a risk factor for SIDS.	51.4	19
	All answers are correct.	16.2	6
	Fulfills 4–5 correct measures.	51.4	19
	Fulfills at least 4 correct measures.	32.4	12
Incorrect			
	During the first days of life the baby sleeps in his parent's bed.	0.81	3
	Sometimes I fall asleep while holding the baby.	16.2	6
	The baby sleeps on his lateral side due to gastroesophageal reflux.	13.5	5
	The baby sleeps with the belly down facing the bed.	0.00	0
	I use a duvet, blankets, pillows, and a bonnet so that the baby is not cold while he is sleeping.	0.00	0
	I have toys and teddy bears in the cradle so that the baby does not feel lonely.	0.00	0
	Regardless of the room's temperature, the baby wears a body, plus trousers and baby-grow to sleep.	29.7	11
	I cover the baby with the blankets up to his neck.	0.54	2
	During the first days I used "baby swaddle" for the baby to sleep.	0.54	2
	When the baby sleeps with the pacifier it has a chain.	0.00	0
	Sometimes I leave the baby sleeping in the baby coke at home.	0.00	0
	My baby sleeps in his room.	0.54	2
	The father smokes at home but not in the baby's room so it's safe.	0.00	0
	I can smoke as long I am not breastfeeding.	0.00	0

## DISCUSSION

The authors were able to obtain a sample of 70 pregnant women willing to participate in the study (about 91% of all who attended the CPCP). Their mean age was 32 years. Regarding education, 90% had at least completed high school which is in concordance with the current law that obliges pupils to finish high school, and with the fact that none of the participants were minors. About 51% of pregnant women had a graduation or master's degree; this is consonant with most studies on the subject,^7,8^ but differs from a similar Portuguese study^9^ in which most of the participants did not hold higher education.

Most women were 32 weeks pregnant. At this gestational age, as the due date approaches, a lot of doubts and insecurities settle down, thus, many pregnant women seek help at this time.

In the pre-intervention questionnaire, the rate of correct answers was about 60.0–84.0%. The question that was answered correctly more times concerned the place where the baby is supposed to sleep, and the question with fewer correct answers was about sleep safety. The alarming factor regarding these results is that, prior to the intervention, about 16–40% of the participants did not know the adequate place and environment for a baby to sleep in. Other authors^7-9^ came to that same conclusion, demonstrating, in their study, that about only half the parents recognized the safest way on how to lay a newborn to sleep. That is, if we extrapolate this data to the general population, a significant number of newborns and infants are globally at risk of SIDS since parents are not aware of the correct position, adequate place, and safest environment for their child to sleep.

After the intervention, the authors obtained significantly better results. In several questions, the correct answer rate was 100%, which confirms the efficacy of the lectures. Furthermore, after it, 28 more participants were able to answer all questions correctly.

As for the questionnaire sent one month after the intervention, only 37 women replied. The median age was similar as well as the education level, which was expected. Although 40.5% of the participants did not select any incorrect measures, only 8.1% complied with all procedures learned during class. This was somewhat disappointing since the authors obtained such excellent results in the questionnaire sent after the CPCP sessions, assuming that the measures would be complied with or followed. The incorrect measure selected more times was "Regardless of the temperature of the room I have worn a body, pants and baby-grow." which reflects one of the most common doubts parents have: what is the adequate amount of clothes a baby must wear. In a similar study,^10^ the authors obtained a 34% reduction in prone positioning in the post-intervention group; regarding other non-prone sleeping positions, they found no statistically significant differences between the two groups. An alarming conclusion of this study was that 40.4% of the mothers stated that they obtained recommendations on prone sleeping position from medical professionals. This aspect could have been explored in our study. Another study^11^ also proved to be efficient in improving parents’ knowledge about SIDS and its preventive measures; in the intervention group, 77% of parents put their babies to sleep in a prone position compared to 44% in the control group. Moreover, in the control group, only 54% of parents used the crib as a place for their baby to sleep in.

Nevertheless, and comparatively with other studies, the authors were able to transmit the correct information to some of the participants who were complying with it, which proves this study to be satisfactory.

Although SIDS is the leading cause of post-neonatal mortality in the first year of life in developed countries, globally, it is relatively uncommon with an incidence of one case per 1,000 infants.^1-3,12^ Its etiology remains unknown. Several studies suggest some preventable risk factors like smoke exposure and sleeping in lateral or ventral decubitus, among others. On the other hand, some protective factors have also been gaining some recognition, such as: breastfeeding, use of pacifiers, sleeping in a cot in a room shared with parents, etc.^3,13,14^ Since the early 1990s, after discovering that sleeping in a prone position tripled the risk of SIDS, sleeping in a supine position has been recommended all over the world.^15^

Other preventive measures have also been recommended. However, through infants’ routine appointments and scientific studies,^7-11^ it was noticed that many parents still aren't familiar with the existence of this syndrome as well as the potential risk and protective factors. Even those who are acquainted with SIDS do not follow entirely and in an adequate fashion the recommendations on how to prevent it. So, with this quasi-experimental before and after intervention study, the authors sought to assess general knowledge about SIDS among pregnant women who attended CPCP at ACeS Ave-Famalicão and the impact of an intervention on their knowledge and compliance.

This intervention study was pioneer in investigating SIDS at a national level, yet some flaws can be highlighted. The authors consider the sample size to be a weakness of the study but, effectively, during the period established for the intervention, only 77 pregnant women attended the course of which only 70 agreed to participate. It is fact that our sample was higher than the calculated size (n=65) required to obtain a significant sample from the population with a 95%CI; however, of all participants, only 64 answered the post-session questionnaire and merely 37 answered the questionnaire sent one month after the intervention, even though they were notified by email. This might be explained by the fact that some participants do not check their email frequently, did not have access to the internet at that time, or were overloaded with newborn care. Another important point to mention is the timeframe between the application of the three questionnaires; this is, the third questionnaire was sent to the participants one month after the intervention. By this time, many of the participants had already given birth so their responses to the questionnaire may have been positively biased due to them being more aware of SIDS and related aspects, through pamphlets and posters present at the maternity ward and advice given by Nurses, Pediatricians, and Family Doctors.

Nonetheless, the authors were able to achieve statistically significant results, although those cannot be extrapolated to the rest of the population. This is because mothers’ knowledge may vary with geographic location, integration, social exposure, and their will to learn more about certain subjects, among other variables, which could be under or even overrated in a small sample like the one in this study.

With this study, the authors concluded that it is possible to improve general knowledge about SIDS, mainly its risks and protective factors, as it can be seen by the improved scores in the post-intervention questionnaire.

However, the authors are also aware that improving knowledge about a subject does not necessarily mean one will change habits and behavior, as revealed in the results of the third questionnaire. Therefore, one session about SIDS is not enough to consolidate knowledge about it; it depends on constant exposure to relevant and essential information on a given topic in order to really be able to impact parents and thus change their newborn care practices.

Hence, since it is a Public Health issue, there is a window of opportunity to effectively promote safe practices and educate new parents or parents-to-be concerning the simple measures needed to prevent SIDS. Therefore, it is imperative to implement a session about SIDS in the CPCP as well as spread awareness through campaigns nationwide in health centers, schools, streets, and even malls.

The newborn's first appointment also plays a leading role in caregivers’ education when health professionals must reinforce preventive measures and avoid risk factors for SIDS.

The authors propose an intervention study, similar to the present study, to be applied at a national range in the future. Considering the disparity between the degree of knowledge and the rate of applicability of the preventive measures, the authors also suggest studying the barriers that foster this gap.

## Data Availability

The database that originated the article is available with the corresponding author.
